# Genome-Wide Identification and Expression Profiling Analysis of the Mitochondrial Calcium Uniporter Gene Family Under Abiotic Stresses in *Medicago sativa*

**DOI:** 10.3390/plants13223176

**Published:** 2024-11-12

**Authors:** Wanhong Li, Bowei Jia, Jiaxun Sheng, Yang Shen, Jun Jin, Xiaoli Sun, Xiangping Liu, Mingzhe Sun

**Affiliations:** 1 Crop Stress Molecular Biology Laboratory, Heilongjiang Bayi Agricultural University, Daqing 163319, China; wh10161122@163.com (W.L.); jiabowei_paper@163.com (B.J.); 17604526492@163.com (J.S.); shenyang7788@byau.edu.cn (Y.S.); jjbyau990421@163.com (J.J.); 2 Grassland Science Laboratory, Heilongjiang Bayi Agricultural University, Daqing 163319, China; 3 Key Laboratory of Soybean Biology of Chinese Education Ministry, Northeast Agricultural University, Harbin 150030, China

**Keywords:** mitochondrial calcium uniporter, *Medicago sativa*, expression analysis, abiotic stress

## Abstract

The mitochondrial calcium uniporters (MCUs) are a family of calcium unidirectional transporters important for cytoplasmic Ca^2+^ signals. Though the MCU proteins in several plant species have been investigated, genome-wide analysis of *MCUs* in alfalfa is lacking. Here, via genome-wide analysis, a total of 5, 20, and 6 *MCU* genes were identified in three different alfalfa cultivars, namely *Medicago truncatula* Jemalong A17, *Medicago sativa* XinJiangDaYe, and *M. sativa* Zhongmu No. 1, respectively. They were further phylogenetically classified into three subfamilies. Most *MCU* genes have only one intron, and gene duplication events of *MCU* genes were observed within each alfalfa accession and between different accessions. All alfalfa MCU proteins contained a highly conserved MCU domain and 10 conserved motifs, featuring two transmembrane domains and a DI/VME motif. According to the tissue expression data of *M. sativa* Zhongmu No. 1, *MsMCU6.2* was the most abundant transcript with the highest expression in the leaf, and *MsMCU5* and *MsMCU1.2* showed higher expression levels in the stem than other tissues. We analyzed the expression profiles of five *MCU* genes (*MsMCU1.1/1.2/5/6.1/6.2*) under salt, drought, and cold stresses via qRT-PCR assays. All five *MCU* genes were induced by drought stress, except MsMCU5, whose expression was up-regulated by salt stress, while cold stress slightly altered MsMCU expression. Nine potential interacting proteins and three miRNAs targeting *MtMCUs* were predicted. These results provide detailed knowledge of alfalfa *MCU* genes and suggest their potential functions in abiotic stress response.

## 1. Introduction

Alfalfa (*Medicago sativa*) is a perennial herbaceous pasture grass widely cultivated to improve grasslands and farmlands. Alfalfa is rich in protein and minerals, which can be used as a source of nutrition for livestock and poultry. In addition, alfalfa can also be used as a soil improvement plant, which helps to improve soil fertility and nitrogen fixation [1].

Extreme weather and climate events, such as heat waves, drought, and cold, occur frequently and globally. Global climate change leads to abiotic stresses, such as cold attacks, water deficit, and soil salinization, which repress plant growth, reduce yields, and finally impact agricultural production. The yield of alfalfa is severely affected by these diverse adverse environmental factors [2,3,4,5,6]. Soil salinity (>0.3%) and drought impair alfalfa seed germination and seedling growth. High salinity in soils increases cytoplasmic ion concentration, causing dehydration of alfalfa cells and decreasing fresh weight in developed plants. Under drought stress, the root system of alfalfa absorbs insufficient water and nutrients, leading to poor growth and development and decreased yield. Moreover, once alfalfa is subjected to low temperatures, physiological metabolism is disrupted, causing plant tissue necrosis. Therefore, mining stress-related genes and exploring their related functions in abiotic stress is of great significance for the genetic improvement of alfalfa.

Calcium is an essential mineral nutrient in plant growth and development. Usually, calcium exists in the form of cation (Ca^2+^) in soil, and plant roots absorb Ca^2+^ into cells through transporters or channels, such as cyclic nucleotide-gated channels (CNGCs), glutamate-like receptors (GLRs), two-pore cation channel (TPC), Mid1-complementing activity channels (MCAs), hyperosmotic pressure-induced [Ca^2+^]cyt-increasing channels (OSCAs), and annexins (ANNs) [7,8]. In plant cells, intracellular Ca^2+^ exists in the “calcium pool”, such as cell wall, endoplasmic reticulum, mitochondria, chloroplasts, vacuoles, and peroxisomes [9,10]. Under normal conditions, cytoplasmic Ca^2+^ concentration is maintained at a stable and low level [9,10,11]. Once stimulated by external factors, the concentration of cytoplasmic Ca^2+^ dramatically increases, referred to as calcium oscillation [12,13].

It has been well demonstrated that cytoplasmic Ca^2+^ is a type of second messenger that triggers different downstream signaling pathways under diverse abiotic stresses. Cytoplasmic Ca^2+^ signals can be perceived by sensors such as CaM/CMLs (calmodulins/CaM-like proteins) [14,15], CBLs (calcineurin B-like proteins) [16], and CDPKs (calcium-dependent protein kinases) [17]. CaM/CMLs and CBLs interact with CCaMK (Ca^2+^/CaM-dependent protein kinases) and CIPKs (CBL-interacting protein kinases) to activate their targets [18,19]. These protein kinases transduce signals by phosphorylating a wide array of substrate proteins to control various physiological and biochemical processes during plant response to abiotic stresses.

Ca^2+^ influx into mitochondria acts as a calcium pool that modulates intracellular calcium signaling transduction. Mitochondrial Ca^2+^ uptake is controlled by the calcium uniporter complex within the mitochondrial membrane [20,21]. MCU is a highly conserved 40 kD protein with Ca^2+^ permeability channel activity and is a central component of the unidirectional calcium ion transporter of the inner mitochondrial membrane [22]. Its homologous genes are reported to be widely distributed in plants, metazoans, and fungi [10,23,24,25]. The MCU protein architecture contains two coiled-coil domains, two transmembrane domains, and a DI/VME motif. The DI/VME motif, located between the two transmembrane domains, consists of negatively charged amino acids, which are potential Ca^2+^-binding sites and are essential for the function of MCU [25].

To date, *MCU* genes have been extensively characterized in *Arabidopsis thaliana* (Arabidopsis) (6 *AtMCU*) [26], *Pyrus seratina* (ground pears) (7 *PbrMCU*) [27], *Sorghum bicolor* (sorghum) (4 *SbMCU*) [28], *Glycine soja* (wild soybean) (11 *GsMCU*) [29]. However, genetic evidence regarding MCU function in alfalfa is lacking. Hence, exploring the stress-responsive genes in alfalfa will be key to breeding new cultivars with improved tolerance to adverse environmental stress. *Medicago truncatula* is a diploid legume plant related to the forage crop alfalfa. The reference genome sequences of three alfalfa varieties have been published, including the haploid genome of *M. truncatula* Jemalong A17 [30] and *M. sativa* XinJiangDaYe [31], and the tetraploid genome of *M. sativa* Zhongmu No. 1 [32]. Taking advantage of the available alfalfa genome sequence, we identified *MCU* family genes in three different alfalfa cultivars and systematically analyzed their phylogenetic relationship, gene architecture, conserved domain, and expression profiles. Our results indicated that alfalfa *MCU* genes were evolutionarily conserved and showed tissue expression specificity. Importantly, we identified MCUs that responded to salt, drought, and cold stress, which will be useful for molecular breeding for new salt-, drought-, and cold-tolerant alfalfa varieties.

## 2. Results

### 2.1. Identification of Mitochondrial Calcium Uniporter Family Members in Alfalfa

**Figure 1:**
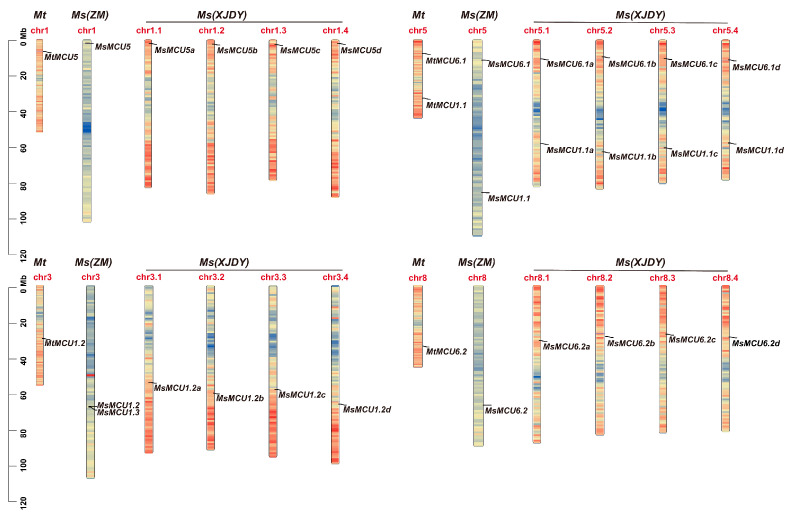
Chromosomal location of the *MCU* family genes in *M. truncatula*, *M. sativa* XinJiangDaYe, and Zhongmu No. 1. Colored columns represent chromosomes, and the grey line indicates the position of each MCU gene. The rainbow color on each chromosome illustrates gene density, with high density in red and low density in blue.

Based on the amino acid sequences of MCU proteins from Arabidopsis [26] and wild soybean [29], we identified the putative MCU proteins in *M. truncatula* Jemalong A17, *M. sativa* XinJiangDaYe, and Zhongmu No. 1. As a consequence, we identified 5 *MCU* genes in *M. truncatula*, 20 in XinJiangDaYe, and 6 in Zhongmu No. 1, respectively (Table S1). They were named based on their homology to *AtMCUs* (Table S1). Chromosomal localization showed that five *MtMCUs* were distributed on four chromosomes, with two genes on chromosome 5 (Figure 1). Four copies of each *MtMCU* were identified in the XinJiangDaYe genome. The corresponding gene of each *MtMCU* was also identified in the Zhongmu No. 1 haploid genome. Notably, we also identified one additional member in Zhongmu No. 1, *MsMCU1.3*, which did not possess the corresponding gene in *M. truncatula* and XinJiangDaYe genome.

As predicted by WoLF PSORT, the localization of MCU proteins is characterized by a dual distribution in mitochondria and chloroplasts. Precisely, MtMCU1.1, MtMCU6.1, MsMCU1.1a/b/d, and MsMCU6.1a/b/d were predicted to be localized in the inner mitochondrial membrane. MtMCU5, MtMCU6.2, MsMCU1.1, MsMCU5, MsMCU1.2a/b/c/d, and MsMCU6.2a/b/d are predominantly localized to the cystoid membranes of chloroplasts (Table S2).

### 2.2. Phylogenetic Analysis of Alfalfa MCU Family

**Figure 2:**
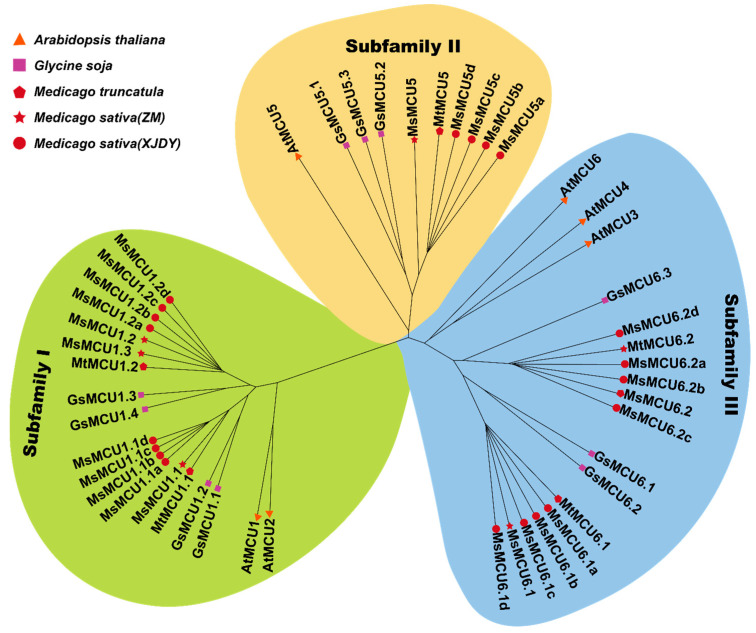
Phylogenetic analysis of the MCU family from alfalfa, wild soybean, and Arabidopsis. The Neighbor-Joining tree was constructed using the protein sequences of MCUs in Arabidopsis (AtMCUs, orange triangle), wild soybean (GsMCUs, purple square), *M. truncatula* (MtMCUs, red five-sided figure), and *M. sativa* (MsMCUs, red five-pointed star for Zhongmu No. 1 and red circle dot for XinJiangDaYe). Different subfamilies were distinguished by different colors; subfamilies I, II, and III are represented by green, yellow, and blue colors, respectively.

Previous studies have shown that six *AtMCUs* were clustered into two major groups, one consisting of AtMCU1 and AtMCU2 and the other consisting of AtMCU3-MCU6 [33,34]. To investigate the phylogenetic relationship among alfalfa MCU family members, we constructed a Neighbor-Joining tree using the full-length amino acid sequences of Arabidopsis, wild soybean, and alfalfa MCU proteins (Figure 2). As shown in Figure 2, these MCU proteins were clearly classified into three subfamilies. In each subfamily, AtMCUs were grouped into a distinct branch and separated from alfalfa and wild soybean homologous MCUs. Subfamily I was separated from the other two subfamilies, which included AtMCU1 and AtMCU2, as well as homologous MCU proteins in alfalfa and wild soybean. Subfamily II contained AtMCU5 and homologous MCU5s. Subfamily III consisted of AtMCU3, AtMCU4, and AtMCU6, as well as homologous MCU6s from alfalfa and wild soybean, indicating potential gene loss during legume evolution. Consistent with the chromosomal location, one MsMCU from Zhongmu No. 1 and four corresponding MsMCUs from XinJiangDaYe were clustered together with the MtMCU in each subfamily, and MsMCU1.3 was adjacent to MsMCU1.2. Together, these results suggest that the MCU family members in the process of evolution are highly conserved.

### 2.3. Gene Structure and Duplication Analysis of Alfalfa MCU Family

**Figure 3:**
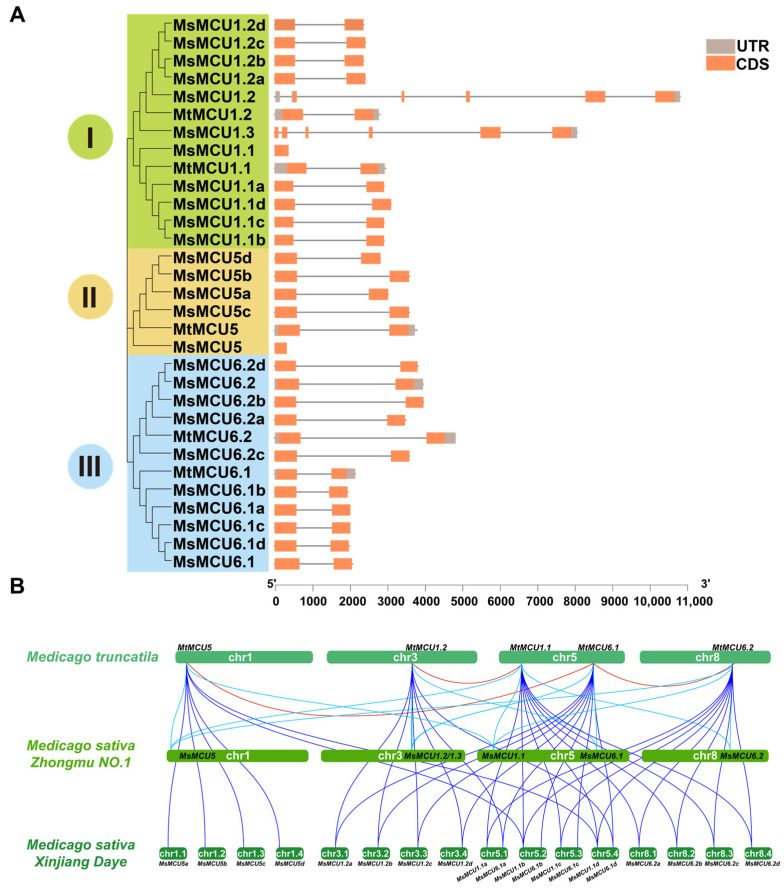
Gene structure and duplication analysis of the alfalfa *MCU* family: (**A**) Intron–exon architecture of alfalfa *MCU* genes. Yellow boxes represented exons, and grey lines represented introns. 5′ and 3′ untranslated regions (UTRs) were represented by grey boxes. The scale of gene length was given at the bottom. CDS: Coding sequence. (**B**) Duplication analysis of alfalfa *MCUs*. Red lines indicated duplication relationships within *M. truncatula*, light blue indicated duplicated pairs between *M. truncatula* and *M. sativa* Zhongmu No. 1, and dark blue indicated duplicated pairs between *M. truncatula* and XinJiangDaYe.

To further understand the alfalfa *MCU* family, we analyzed their intron–exon organization (Figure 3A). It was observed that all *MtMCU* and most *MsMCU* genes contained a single intron. *MsMCU1.2* and *MsMCU1.3* from Zhongmu No. 1 contained five introns, while *MsMCU1.1* and *MsMCU5* contained only one exon, which might be due to incomplete sequences. The UTRs (untranslated region) exist at both 5′ and 3′ ends in all *MtMCUs* but are missing in most *MsMCUs.* Specifically, there was no UTR in *MsMCUs* from XinJiangDaYe.

Duplication analysis revealed three duplicated pairs of *MtMCU* genes, including *MtMCU1.1/1.2*, *MtMCU6.1/6.2,* and *MtMCU5/6.1* (Figure 3B). Furthermore, there were 10 duplicated MCU pairs between *M. truncatula* and *M. sativa* Zhongmu No. 1 and 36 pairs between *M. truncatula* and *M. sativa* XinJiangDaYe. In addition, *MsMCU1.1/1.2*, *MsMCU6.1/6.2*, and *MsMCU5/6.1* also have duplication relationships, which may be caused by fragment duplication. In summary, these duplication events indicate high conservation in the gene structure of the alfalfa *MCU* family.

### 2.4. Protein Conservation Analysis of the MCU Family in Alfalfa

**Figure 4:**
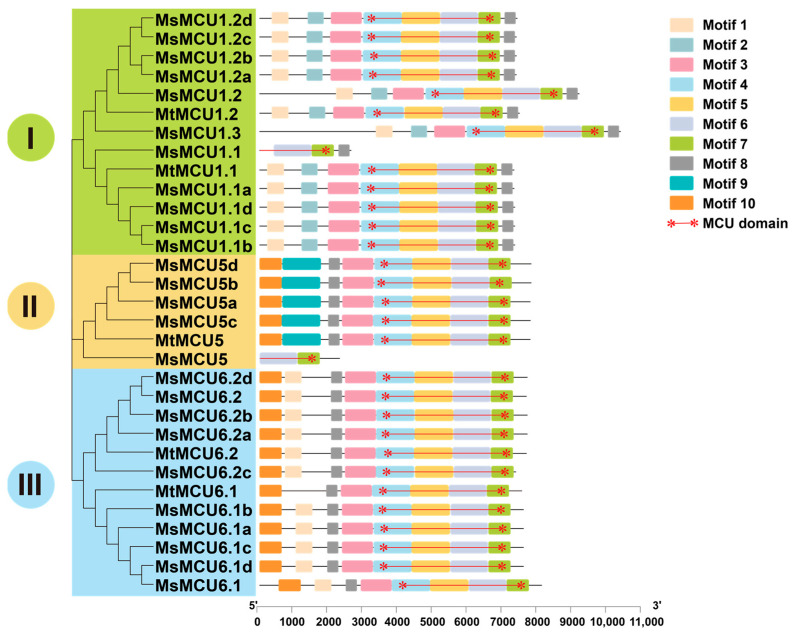
Conserved domains and motifs of alfalfa *MCU* family proteins. Grey lines indicated protein sequences, and the color boxes represented conserved motifs. The combination of red asterisks and lines represented the conserved MCU domains. The scale of protein length was given at the bottom.

To further investigate the conservation of the alfalfa *MCU* family, we analyzed their protein structure. All MCU proteins in alfalfa contained a complete MCU functional domain (PF04678) near the C-terminus, except the incomplete MCU domain in MsMCU1.1 and MsMCU5 (Figure 4). The difference in exon–intron structure of *MsMCU1.1* and *MsMCU5* suggested that their incomplete genomic sequences were possibly due to the quality of the *M. sativa* Zhongmu No. 1 genome. As shown in Supplemental Figure S2, the MCU domain consisted of three coil–coil helix domains (CCH1, CCH2a, and CCH2b) and two transmembrane helices (TMH1 and TMH2), with an additional N-terminal extension domain (NTD). Furthermore, multiple sequence alignment confirmed the high conservation of a DI/VME motif and two amino acids, Arg and Glu, located between TMH1 and TMH2, which were essential for Ca^2+^ transport activity [35,36] (Supplemental Figure S1). To support the structural conservation of alfalfa MCU proteins, we calculated the sequence identity of each MCU pair. As shown in Supplemental Figure S2, protein sequence identity within each subfamily ranged from 55% to 100%. The similarity of protein sequences between subfamily II and III members was especially high compared to that of subfamily I. This result is consistent with the close relationship between subfamily II and III in the phylogenetic tree.

### 2.5. Expression Levels and Variance Analysis of Alfalfa MCU Gene

**Figure 5:**
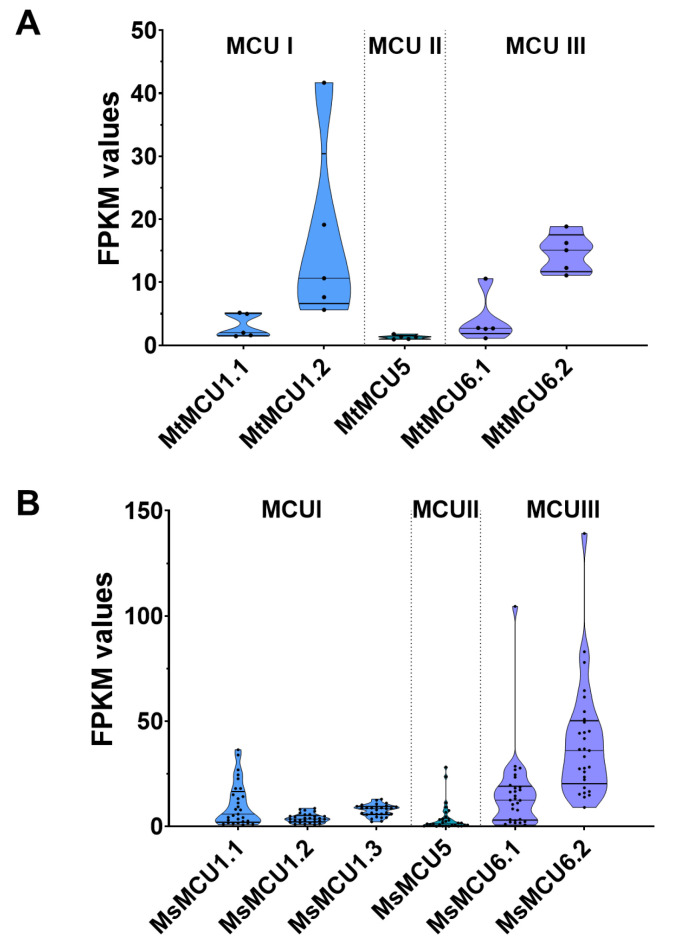
Expression values and variance of the MCU genes in M. truncatula (**A**) and M. sativa Zhongmu No. 1 (**B**). Black dots in violin plots stand for FPKM (Fragments Per Kilobase per Million) values of each MCU gene. The three horizontal lines inside the violin plots represent the first interquartile range (IQR), the median, and the third IQR, respectively.

To evaluate the expression of *MCU* genes, we retrieved the public transcriptome data and analyzed their expression profiles in *M. truncatula* and *M. sativa* Zhongmu No. 1. The violin plots showed that *MtMCU1.2* and *MtMCU6.2* showed relatively higher expression levels, with greater expression variance of *MtMCU1.2* (Figure 5A). In *M. sativa* Zhongmu No. 1, the expression of *MsMCU6.1* and *MsMCU6.2* from subfamily III displayed much higher levels and greater variance than other members (Figure 5B). In detail, *MCU5* exhibited the lowest expression levels in both *M. truncatula* and *M. sativa* Zhongmu No. 1. Differently, *MCU1.2* showed higher expression with greater variance in *M. truncatula*, but lower expression and smaller variance was observed in *M. sativa* Zhongmu No. 1. These findings indicate that even though they share high sequence conservation, *MCU* genes displayed distinct expression patterns in *M. truncatula* and *M. sativa*.

### 2.6. Expression Patterns of MsMCU Genes in Different Tissues

**Figure 6:**
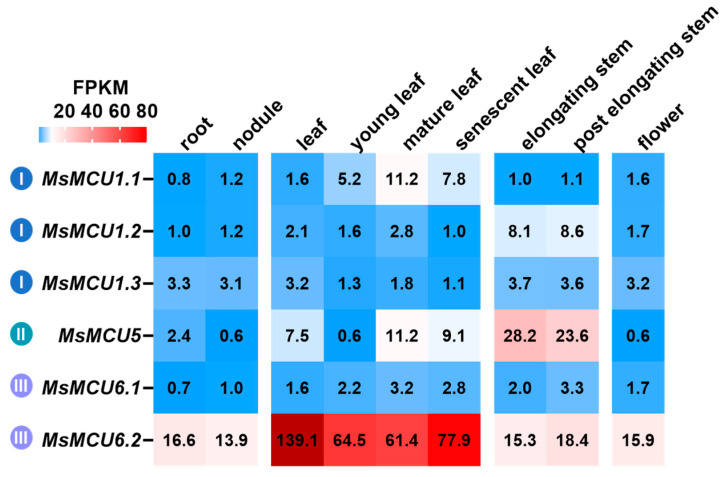
Expression of the *MCU* and genes in different tissues of *M. sativa* Zhongmu No. 1. Numbers on the heatmap represent the FPKM values of each gene based on the transcriptome data. Roman numerals in colored circle dots represent different subfamilies of the MCU family.

We further investigated the expression patterns of *MsMCU* genes in different tissues of Zhongmu No. 1, including root, nodule, leaf, young leaf, mature leaf, senescent leaf, elongating stem, post-elongating stem, and flower [37]. As depicted in Figure 6, among six *MsMCUs*, *MsMCU6.2* was the most abundant transcript, with the highest expression levels in leaves. *MsMCU1.1* expression also showed higher levels in leaves, suggesting the possible involvement of *MsMCU6.2* in alfalfa growth and development. Moreover, *MsMCU5* and *MsMCU1.2* showed higher expression levels in stems, indicating their potential role in the development or lignification of the stem. *MsMCU1.3* and *MsMCU1.2* expression were maintained at roughly similar levels in different tissues. These data indicate potential different functions of the MCU family and point out possible functional redundancy of certain *MCUs* with overlapping tissue expression.

### 2.7. Expression Patterns of MsMCUs Under Different Environmental Conditions

**Figure 7:**
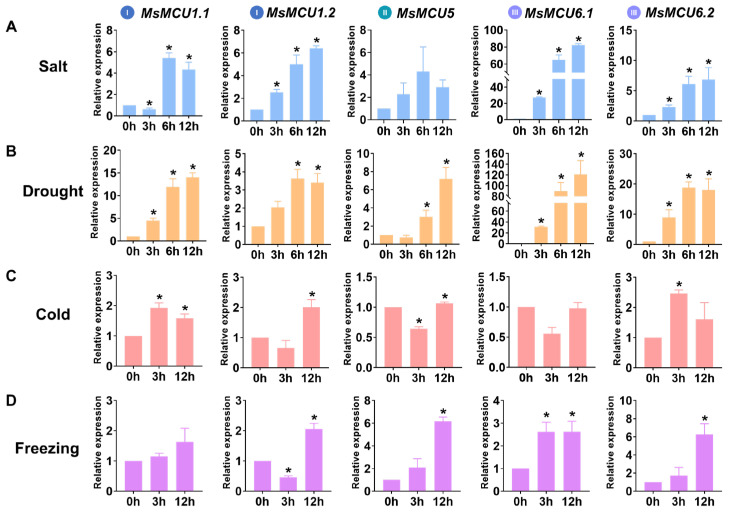
Quantitative real-time PCR assays of *MsMCUs* under abiotic stresses in *M. sativa* Zhongmu No. 1. (**A**–**D**), qRT-PCR assays of *MsMCUs* under salt (250 mmol/L NaCl), drought (400 mmol/L mannitol), cold (4 °C), and freezing (−5 °C) treatments. Columns and bars represent the means and standard deviation from three biological replicates. The expression at 0 h was set as 1, and asterisks indicate statistically significant differences compared with 0 h by the Student’s *t*-test (* *p* < 0.05). Roman numerals in colored circle dots represent different subfamilies of the MCU family.

We further analyzed *MsMCUs* gene expression under salt, drought, and cold stress using the RNA-seq data of Zhongmu No. 1 [4,38,39]. As shown in Supplemental Figure S3, except for *MsMCU1.3*, other *MsMCUs* were differentially expressed under both salt and drought stresses, as evidenced by the |Log_2_FC| values ≥ 1. Compared with salt and drought stresses, only *MsMCU1.1* was down-regulated by cold stress. We further carried out quantitative real-time PCR (qRT-PCR) to investigate the stress response mechanisms of *MsMCUs*. It was observed that only *MsMCU1.3* showed no changes in expression based on the public transcriptome data. Subfamily I members *MsMCU1.1* and *MsMCU1.2* were dramatically up-regulated by salt, drought, and cold stress (Figure 7). *MsMCU5* from Subfamily II was significantly induced by drought and cold stresses but not by salt stress. *MsMCU6.1* and *MsMCU6.2* were dramatically induced by salt and drought stresses. After cold treatment for 3 h, *MsMCU6.1* overexpression was decreased to half, while *MsMCU6.2* was slightly up-regulated (Figure 7C). The changing folds under cold stress were much smaller than those under salt and drought stress (Figure 7, Supplemental Figure S3). One possible explanation is that alfalfa is more tolerant to low temperatures than other plants because of the overwintering habit. Therefore, we further checked the expression changes under freezing (−5 °C) stress (Figure 7D). Except for *MsMCU1.1*, other *MsMCUs* showed increased expression levels after freezing treatment. In summary, these results suggest that *MsMCUs* were involved in abiotic stress response.

### 2.8. Prediction of Proteins and miRNAs Interacting with MtMCUs

**Figure 8:**
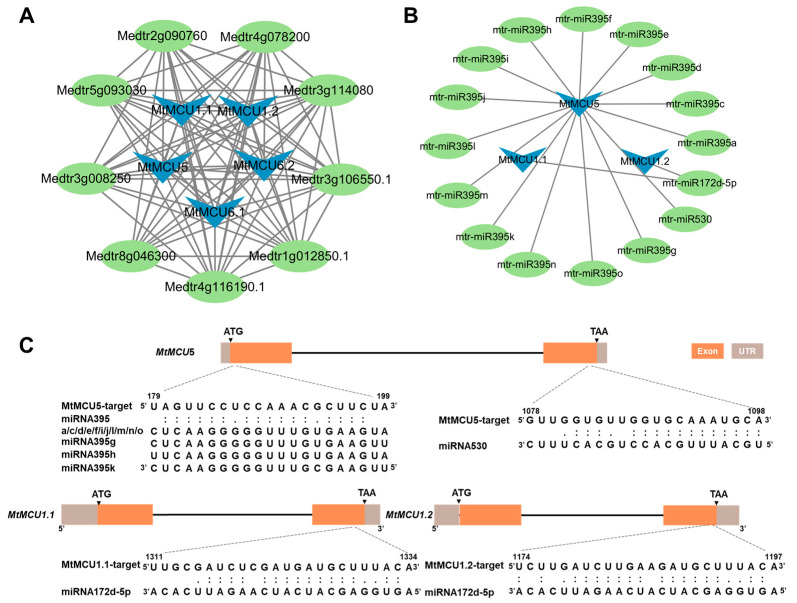
Prediction of proteins and miRNAs interacting with MtMCUs: (**A**) Proteins interaction analysis of MtMCUs. (**B**) miRNAs interaction analysis of *MtMCUs.* (**C**) Schematic of *miRNA* target sites in *MtMCUs* transcript.

The protein–protein interaction prediction identified nine interactive partners of MtMCUs, including three calcium-binding EF hand proteins (Medtr3g106550, Medtr4g116190, Medtr1g012850), one Zinc-finger FYVE domain-containing protein (Medtr3g114080), and five Prohibitin (Medtr4g078200, Medtr2g090760, Medtr8g046300, Medtr3g008250, Medtr5g093030) (Figure 8A, Table S3). Notably, all of these nine partners were predicted to interact with each MtMCU protein, forming 81 interacting pairs. This finding further supports the conservation of the MCU family and highlights the potential regulation of MCU transport activity via protein–protein interaction.

Prediction of potential miRNAs showed that 14 members of the *miR395* family could target *MtMCU5* (Table S4). Although there are minor differences among these mature miR395 sequences, they targeted the same position of *MtMCU5*, located in the first exon. *MiR530* also targeted *MtMCU5* at the second exon. A 24 nt miRNA, *MiR172d-5p*, targeted both *MtMCU1.1* and *MtMCU1.2* at the second exon. These findings suggest potential post-transcriptional regulation of *MCU* expression in alfalfa.

## 3. Discussion

MCU is a calcium transport protein, which is the primary mediator of calcium ion entry into the mitochondrial matrix [40]. It was first identified in mammals as a senescence regulator, but its role in plant cells is poorly understood. Phylogenetic analysis of *MCU* family genes from 36 plant species suggested that most species have three or more MCU homologs, except for only one copy in *Chlamydomonas reinhardtii* and *Volvox carteri* [34]. The numbers of *MCU* genes vary significantly among different plant species. For example, there are 6 *AtMCUs* in Arabidopsis [10], 6 *ZmMCUs* in maize (*Z. mays*) [24], 7 *PbrMCUs* in pear (*P. bretschneideri*) [27], and 11 *GsMCUs* in wild soybean [29]. Among them, soybean has more MCU genes because soybean, as a typical ancient polyploid, underwent two rounds of whole genome duplication events [41]. In this study, we identified 5 *MtMCU* genes, 6 *MsMCUs* in *M. sativa* Zhongmu No. 1, and 20 *MsMCUs* in *M. sativa* XinJiangDaYe (Table S1 and Figure 1). The *MCU* number in XinJiangDaYe was about four times that in *M. truncatula* and *M. sativa* Zhongmu No. 1 because the reference genome of *M. truncatula* and *M. sativa* Zhongmu No. 1 used in this study are haploid, while the XinJiangDaYe genome is autotetraploid [31,42]. This finding was supported by a previous report of the *HSF* (heat shock factor) family, with 16 members in *M. sativa* Zhongmu No. 1 [43] and 104 in XinJiangDaYe [44]. Similarly, higher numbers of Ca^2+^ transporter genes, such as CaCA (Ca^2+^/cation antiporters) and OSCA (hyperosmolality-induced [Ca^2+^] increase) (two-pore K^+^ channels), have also been reported in allohexaploid *Triticum aestivum* (bread wheat) [45,46,47].

In Arabidopsis, MCU proteins were phylogenetically clustered into two subfamilies: subfamily I consisted of two closely related MCUs (AtMCU1 and AtMCU2), and subfamily II consisted of the other four members (AtMCU3-AtMCU6). In soybean, the MCU family was divided into subfamily I (containing four MCU1 homologs) and subfamily II (consisting of three MCU5 and four MCU6 homologs); therefore, subfamily II was further divided into two branches, namely the MCU5 and MCU6 branch, respectively. This evolutionary relationship may originate from gene replication [26,29]. In this study, we classified these 31 alfalfa *MCU* genes into three subfamilies (Figure 2). Subfamilies II and III clustered together and separated from subfamily I (Figure 2). Consistently, gene duplication analysis (Figure 3) showed that *MtMCU6.1* (subfamily III) was paralogous to *MtMCU5* (subfamily II) and *MtMCU6.2* (subfamily III); *MtMCU1.1* (subfamily I) was the duplicated gene of *MtMCU1.2* (subfamily I). Furthermore, *MtMCU6s* were orthologs of *MtMCU6.1* and *MtMCU6.2*; *MsMCU5s* were orthologous genes of *MtMCU5*, and *MsMCU1s* were orthologous genes of *MtMCU1.1* and *MtMCU1.2* (subfamily I) (Figure 3). These results indicated that the expansion of XinJiangDaYe *MsMCU* was due to the chromosome doubling [31].

Structurally, *MCU* members with close phylogenetic relationships showed similar results. In terms of exon–intron architecture, most alfalfa *MCU* genes contained two exons of similar length and one long intron (Figure 3A). Similar exon–intron composition was observed for *MCU* genes in maize [24], soybean [29], and rice (*Orza sativa*) [29]. In terms of protein structure, Arabidopsis MCU proteins consisted of a mitochondrion-targeting signal (MTS), an N-terminal domain (NTD), two coiled-coil domains (CCH1 and CCH2), a DV/IME motif, and two transmembrane regions (TM1 and TM2). These featured structures were observed in all alfalfa MCU proteins (Figure 4, Supplemental Figure S2) [22,25,33,48]. Both the N- and C-terminus of MCU proteins face the mitochondrial matrix. The NTD is crucial for MCU transporter activity because overexpression of NTD-lacking MCUs has a significant negative impact on mitochondrial Ca^2+^ uptake [49]. Previous studies found that mutations in the DV/IME motif would lead to the loss of MCU function [50]. Therefore, the highly conserved structure of plant MCU proteins indicates the functional conservation of the *MCU* genes during evolution.

Calcium ion (Ca^2+^) is an important plant signal involved in plant growth, development, and stress response [11]. The MCU proteins are a major contributor to Ca^2+^ uptake in mitochondria [11]. Previous studies reported unchanged mitochondrial Ca^2+^ uptake in roots of *atmcu1* knockout mutants but much lower Ca^2+^ uptake levels in *atmcu123* triple mutants, suggesting functional redundancy of *AtMCU1/2/3* [33,34]. Possibly because all three of these genes were expressed in roots, the functional redundancy could be caused not only by high conservation in protein sequences and structures but also by *AtMCU1/2/3* expression in roots [33]. In wild soybean, duplicated pairs, for example, *GsMCU1.3*-*GsMCU1.4* and *GsMCU6.2*-*GsMCU6.3*, also showed similar tissue expression patterns [29]. In Zhongmu No. 1, *MsMCU1.1* and *MsMCU6.2* had higher expression in leaves, *MsMCU1.2* and *MsMCU5* expression was higher in stems, and *MsMCU1.3* and *MsMCU6.1* did not show distinctive tissue specificity (Figure 6). Future loss-of-function studies of *MsMCU* should consider the function redundancy and tissue expression specificity.

The involvement of *MCU* genes in abiotic stress response has been reported in a few studies. In Arabidopsis, expression of *AtMCU4/6* was induced by salt, drought, and highlight stresses, while *AtMCU2/6* expression was suppressed by high temperature [51]. *AtMCU6* was reported to regulate the MAPK3/6-ERF6-MYB60 signal pathway to control stomatal movement under osmotic stress by mediating chloroplast Ca^2+^ concentration [35]. In this study, except for *MsMCU5*, all *MsMCUs* were significantly induced by drought and salt stresses (Figure 7 and Supplemental Figure S2). Similarly, previous studies have shown that *GmMCU1.1* is down-regulated under drought stress, while *GmMCU1.2* is significantly up-regulated under salt and drought stress [52]. Furthermore, changing folds of *MsMCUs* under cold stress were smaller than those under salt, drought, and freezing stresses (Figure 7), possibly due to alfalfa overwintering habit and greater tolerance to low temperature. Only a few *MCUs* have been functionally characterized in abiotic stress, so further genetic approaches, such as overexpression or knockout, should be performed to clarify their function in stress response. Moreover, considering the fact that stress responses can evolve, longitudinal data will yield insights into adaptive mechanisms. In the future, stress-responsive expression of *MCUs* should be studied in details, for instance under long-term stress, stresses occurring at differing growth stages, or successive stress cycles.

In plants, MCU proteins are suggested to form a complex with MICU (mitochondrial calcium uptake) proteins, which are characterized by two calcium-binding EF-hand domains [53]. AtMICU negatively affected AtMCU1 activity in vitro, and *atmicu* mutants accumulated more Ca^2+^ in mitochondria compared with the wild-type control [53]. Here, we predicted three calcium-binding EF-hand proteins (Medtr3g106550, Medtr4g116190, Medtr1g012850) as interactive proteins of MtMCUs (Figure 8). Furthermore, a zinc-finger FYVE domain-containing protein (Medtr3g114080) was also predicted to interact with MtMCUs (Figure 8). In Arabidopsis, *FYVE1* is essential for plant growth and development, and its mutants are defective in ubiquitin-mediated protein degradation, vacuolar transport, and autophagy [54]. Moreover, *fyve1* knockdown alleles are hypersensitive to ABA, and FYVE1 interacts with and mediates the delivery of the PYL4 ABA receptor to the vacuolar degradation pathway [54,55]. Five PHB (Prohibitin) family proteins (Medtr4g078200, Medtr2g090760, Medtr8g046300, Medtr3g008250, Medtr5g093030) were also potential interacting partners of MtMCUs (Figure 8). *AtPHB3* knockout caused mitochondrial swelling and severe growth retardation [56]. *AtPHB2* overexpression improved salt tolerance [57]. These interacting proteins support the potential roles of MtMCUs in growth and development, as well as abiotic stress response.

We also predicted three miRNAs (*miR395*, *miR172d-5P*, and *miR530*) in alfalfa that could possibly target *MtMCUs* (Figure 8). The *miR395* family and *miR530* target *MtMCU5* but not other *MtMCUs*. Even though the function of these *miRNAs* in alfalfa has not been reported, their homologs in other plant species have been functionally characterized. *OsmiR395* targeted and suppressed the expression of two sulfate transporters, *OsSULTR1* and *OsSULTR2*, which could promote sulfate accumulation and broad-spectrum resistance to bacterial pathogens [58]. *OsmiR530* is suggested to be a negative regulator of grain yield [59]. *miR172d-5p* targets both *MtMCU1.1* and *MtMCU1.2* (Figure 8). *miR172* was involved in developmental transitions [60], flowering time, and floral organ identity [61,62,63]. Although further experiments, including 5′RACE and expression analysis, should be performed to identify the targeting of miRNA-MtMCUs, these findings provide clues to investigate the potential regulatory roles of the miRNA-MtMCUs pairs.

In summary, genome-wide analysis in this study provides insights into the potential regulation of *MsMCU* in abiotic stress tolerance. Considering the impact of frequent extreme weather on alfalfa cultivation, it will be a key point for farmers to select alfalfa cultivars with better drought and salt tolerance. Future work will apply biotechnology and genetic engineering methods to manipulate *MCU* gene expression in alfalfa toward engineering crops with optimized drought or salt tolerance traits.

## 4. Materials and Methods

### 4.1. Identification and Characterization of Alfalfa MCU

The protein sequences of published *MCU* genes in *A. thaliana* and *G. soja* were used to construct a Hidden Markov Model using HMMER [64]. Then, this model was used to search against the proteome database of *M. truncatula* Jemalong A17, *M. sativa* XinJiangDaYe, and *M. sativa* Zhongmu No. 1. The *M. truncatula* proteome sequence was downloaded from the Phytozome database (https://phytozome-next.jgi.doe.gov/info/Mtruncatula_Mt4_0v1, accessed on 6 July 2023) [65]. The proteome sequence of *M. sativa* XinJiangDaYe and *M. sativa* Zhongmu No. 1 were obtained from the MODMS database (https://modms.lzu.edu.cn/, accessed on 14 July 2023) [37]. The Pfam (http://pfam.xfam.org/), SMART (http://smart.embl-heidelberg.de/), and CD-search (https://www.ncbi.nlm.nih.gov/Structure/cdd/wrpsb.cgi, accessed on 21 July 2023) were further applied to confirm the presence of the MCU domain (PF04678). Their potential subcellular localization was predicted by WoLF PSORT (https://wolfpsort.hgc.jp/).

### 4.2. Chromosomal Location and Syntenic Analysis of Alfalfa MCUs

According to the alfalfa genome annotation, the gene density and MCU positions on the alfalfa chromosome were obtained. The duplication events of MCUs were analyzed by MCScanX software (2022) [66]. The syntenic relationship between varieties was analyzed by MODMS (https://modms.lzu.edu.cn/) [37]. The Ka/Ks Calculator function of TBtools was used to calculate non-synonymous alternatives (Ka), synonymous alternatives (Ks), and their ratios [50].

### 4.3. Multiple Sequence Alignment and Phylogenetic Analysis of Alfalfa MCUs

Multiple sequence alignment was performed by Jalview [67] using the protein sequences of MCU in *A. thaliana*, *G. soja*, *M. truncatula*, *M. sativa* XinJiangDaYe, and *M. sativa* Zhongmu No. 1, respectively. A Neighbor-Joining tree was constructed using MEGA7 software (7.0) with 1000 bootstrap replicates.

### 4.4. Gene Structure and Conserved Domain Analysis of Alfalfa MCU

Based on the *MCU* gene sequences of alfalfa, TBtools was used to visualize their exon–intron structure. The MEME website (http://meme-suite.org/tools/meme) was used to analyze the conserved motifs of *MCU* genes and visualize them using TBtools. The number of motifs was set to 10.

### 4.5. Expression Analysis of MCU Genes During Alfalfa Development and Abiotic Stresses Response

The transcript data of *MsMCU* in nine different tissues (root, nodule, leaf, young leaf, mature leaf, senescent leaf, elongating stem, post-elongating stem, and flower) were obtained from the MODMS website (https://modms.lzu.edu.cn/). Expression data of *MsMCUs* under cold (4 °C, seedlings), drought (400 mmol/L mannitol, roots), and salt (200 mmol/L NaCl, roots) treatments (SRR7091780-SRR7091794 and SRR7160313-SRR7160357) were downloaded from NCBI. These data were calculated using Excel 2020, and heatmaps were represented using TBtools (v2.056).

### 4.6. Prediction of Protein and miRNAs Interaction with MtMCUs

The protein-interacting network of MtMCUs was generated using the STRING database (http://stringdb.org/) [68]. The miRNAs-interacting network of MtMCUs was generated using the psRNATarget (16 October 2024), and the maximum threshold for base matching was set to 3.5 [69].

### 4.7. Quantitative Real-Time PCR Analysis of Alfalfa MCU Gene Expression Under Stress Treatment

Seeds of *M. sativa* Zhongmu No. 1 were sterilized using 30% NaClO solution and placed in a lighted incubator for germination. Once cotyledons were fully expanded, seedlings were transferred into either a hydroponic box with 1/4 Hoagland or a vermiculite-filled pot. Seedlings were grown in a growth chamber at 22 °C, with 16 h light/8 h dark photoperiod, 5000 lux light intensity, and 70% humidity. The 1/4 Hoagland solution in the hydroponic box was changed every three days, and the vermiculite-cultured seedlings were irrigated with 1/4 Hoagland solution every three days.

After two weeks, alfalfa seedlings with similar growth performance were selected for stress treatments. For salt and drought treatments, roots of hydroponic alfalfa seedlings were submerged in 1/4 Hoagland solution containing either 250 mmol/L NaCl (salt) or 400 mmol/L mannitol (drought). For cold treatment, vermiculite-cultured alfalfa seedlings were placed in a 4 °C refrigeration incubator. For freezing stress, vermiculite-cultured alfalfa seedlings were treated as described in Supplemental Figure S4. Equal leaves from the same position of the seedlings were sampled at 0, 3, 6, and 12 h, respectively. To exclude the possible circadian influence on gene expression, we started the treatments at different time points to ensure the samples were collected at a unified time point.

Then, these leaves were ground into powder and immersed in TRIZOL to extract total RNA, which was further used to synthesize cDNA using the HiScript III RT SuperMix for qPCR (+gDNA wiper). These cDNA products were then used as templates for qPCR amplification of candidate genes. The reference gene was alfalfa *Actin* (*MsG0380016789*). Primers were designed using Primer 5.0 software. The sequences of primers are listed in Table S5. Gene relative expression levels were calculated using the 2^−∆∆Ct^ method [70]. The expression level at 0 h was set to 1. Three independent biological replicates and three technical repeats were carried out.

### 4.8. Statistical Analysis

Statistical data analysis was performed using the Student’s *t*-test (two-tailed test) with GraphPad Prism9. Statistically significant differences were considered at *p* < 0.05.

## 5. Conclusions

In this study, 5, 6, and 20 *MCU* genes were identified in *M. truncatula, M. sativa* Zhongmu No. 1, and XinJiangDaYe, respectively. Gene duplication was detected within the *MCU* family, and the duplicated pairs displayed a similar exon–intron architecture. Alfalfa MCU proteins were highly conserved in protein sequences, structures, and phylogenic relationships. *MsMCUs* were expressed in different tissues of alfalfa plants and responded to salt, drought, cold, and freezing stresses. This study comprehensively describes the first genome-wide characterization of the *MCU* gene family in alfalfa, and our work will be helpful in facilitating further research on the *MCU* gene family, particularly regarding their evolutionary history and biological functions. Future work will engage with biotechnology and genetic engineering perspectives to manipulate MCU gene expression in alfalfa and create drought or salt-resistant alfalfa cultivars.

## Data Availability

The datasets generated during and/or analyzed during the current study are available from the corresponding author upon reasonable request.

## Supplementary Materials

The following supporting information can be downloaded at: https://www.mdpi.com/article/10.3390/plants13223176/s1. Table S1: The *MCU* family genes in Arabidopsis and alfalfa; Table S2: Prediction of sub-cellular localization of alfalfa MCU proteins; Table S3: Prediction of interacting proteins of MtMCUs; Table S4: Prediction of interacting miRNAs of MtMCUs; Table S5: The primers used in this study. Figure S1: Sequence alignment showing the conserved structure of MCU proteins; Figure S2: Heatmap showing the similarity of MCUs proteins; Figure S3: Expression patterns of MsMCUs in response to salt, mannitol, and cold stresses; Figure S4: Treatment methods for alfalfa seedlings under freezing stress.
