# Supplementary material for: Genome-Wide Identification and Expression Profiling Analysis of the Mitochondrial Calcium Uniporter Gene Family Under Abiotic Stresses in Medicago sativa
Source: Plants (Basel). 2024 Nov 12;13(22):3176. doi: 10.3390/plants13223176 (PMC11598098; doi:10.3390/plants13223176)
Supplement: Supplementary file 1 [file plants-13-03176-s001.zip › plants-3271902-supplementary.pdf]

Table S1 | The *MCU* family genes in Arabidopsis and alfalfa

Plant	Gene Name	Gene ID
Arabidopsis thaliana	*AtMCU1*	*AT1G09575*
	AtMCU2	*AT1G57610*
	AtMCU3	*AT2G23790*
	AtMCU4	*AT4G36820*
	AtMCU5	*AT5G42610*
	AtMCU6	*AT5G66650*
Medicago truncatula	*MtMCU1.1*	*Medtr5g076060*
	MtMCU1.2	*Medtr3g063050*
	MtMCU5	*Medtr1g021955*
	MtMCU6.1	*Medtr5g019350*
	MtMCU6.2	*Medtr8g078170*
Medicago sativa Zhongmu No.1	*MsMCU1.1*	*MsG0580028752.01*
	MsMCU1.2	*MsG0380015087.01*
	MsMCU1.3	*MsG0380015118.01*
	MsMCU5	*MsG0180000180.01*
	MsMCU6.1	*MsG0580024923.01*
	MsMCU6.2	*MsG0880045757.01*
Medicago sativa Xinjiang Daye	*MsMCU1.1a*	*MS.gene017268*
	MsMCU1.1b	*MS.gene99090*
	MsMCU1.1c	*MS.gene009623*
	MsMCU1.1d	*MS.gene26228*
	MsMCU1.2a	*MS.gene008403*
	MsMCU1.2b	*MS.gene049620*
	MsMCU1.2c	*MS.gene55689*
	MsMCU1.2d	*MS.gene05094*
	MsMCU5a	*MS.gene24313*
	MsMCU5b	*MS.gene27343*
	MsMCU5c	*MS.gene26933*
	MsMCU5d	*MS.gene062714*
	MsMCU6.1a	*MS.gene047742*
	MsMCU6.1b	*MS.gene010458*
	MsMCU6.1c	*MS.gene89861*
	MsMCU6.1d	*MS.gene59324*
	MsMCU6.2a	*MS.gene76348*
	MsMCU6.2b	*MS.gene011932*
	MsMCU6.2c	*MS.gene87402*
	MsMCU6.2d	*MS.gene28492*

Table S2 | Prediction of sub-cellular localization of alfalfa MCU proteins.

**Gene Name**	**Predicted location**
MtMCU1.1	Mitochondrial inner membrane
MtMCU1.2	Chloroplast
MtMCU5	Chloroplast thylakoid membrane
MtMCU6.1	Mitochondrial inner membrane
MtMCU6.2	Chloroplast thylakoid membrane
MsMCU1.1	Chloroplast thylakoid membrane
MsMCU1.2	Cytoplasmic
MsMCU1.3	Chloroplast
MsMCU5	Chloroplast thylakoid membrane
MsMCU6.1	Chloroplast stroma
MsMCU6.2	Chloroplast
MsMCU1.1a	Mitochondrial inner membrane
MsMCU1.1b	Mitochondrial inner membrane
MsMCU1.1c	Cytoplasmic
MsMCU1.1d	Mitochondrial inner membrane
MsMCU1.2a	Chloroplast
MsMCU1.2b	Chloroplast
MsMCU1.2c	Chloroplast
MsMCU1.2d	Chloroplast
MsMCU5a	Chloroplast thylakoid membrane
MsMCU5b	Chloroplast thylakoid membrane
MsMCU5c	Chloroplast thylakoid membrane
MsMCU5d	Chloroplast thylakoid membrane
MsMCU6.1a	Mitochondrial inner membrane
MsMCU6.1b	Mitochondrial inner membrane
MsMCU6.1c	Mitochondrial inner membrane
MsMCU6.1d	Mitochondrial inner membrane
MsMCU6.2a	Chloroplast
MsMCU6.2b	Chloroplast
MsMCU6.2c	Chloroplast
MsMCU6.2d	Chloroplast

Table S3 | Prediction of interacting proteins of MtMCUs

Interacting partner	Functional annotation	Arabidopsis homologous	Function
Medtr3g106550	Calcium-binding EF hand protein	AT4G32060	Calcium uptake protein 1 (AtMICU1)
Medtr4g116190	Calcium-binding EF hand protein	AT4G32060	
Medtr1g012850	Calcium-binding EF hand protein	AT4G32060	
Medtr3g114080	Putative chromatin regulator PHD family	AT1G20110	FYVE zinc finger superfamily protein (AtFYVE1)
Medtr4g078200	Prohibitin	AT4G28510	Prohibitin-1 (AtPHB1)
Medtr2g090760	Prohibitin	AT4G28510	
Medtr3g008250	Prohibitin	AT5G40770	Prohibitin-3 (AtPHB3)
Medtr5g093030	Prohibitin	AT5G40770	
Medtr8g046300	Prohibitin	AT5G40770	

Table S4 | Prediction of interacting miRNAs of MtMCUs

miRNA	Target	Expectation	miRNA aligned fragment	Alignment	Target aligned fragment	Inhibition	Position
mtr-miR395a	MtMCU5	3.5	AUGAAGUGUUUGGGGGAACUC	::::::.::::::.::::	UAGUUCCUCCAAACGCUUCUA	Cleavage	179-199
mtr-miR395c	MtMCU5	3.5	AUGAAGUGUUUGGGGGAACUC	::::::.::::::.::::	UAGUUCCUCCAAACGCUUCUA	Cleavage	179-199
mtr-miR395d	MtMCU5	3.5	AUGAAGUGUUUGGGGGAACUC	::::::.::::::.::::	UAGUUCCUCCAAACGCUUCUA	Cleavage	179-199
mtr-miR395e	MtMCU5	3.5	AUGAAGUGUUUGGGGGAACUC	::::::.::::::.::::	UAGUUCCUCCAAACGCUUCUA	Cleavage	179-199
mtr-miR395f	MtMCU5	3.5	AUGAAGUGUUUGGGGGAACUC	::::::.::::::.::::	UAGUUCCUCCAAACGCUUCUA	Cleavage	179-199
mtr-miR395g	MtMCU5	2.5	UUGAAGUGUUUGGGGGAACUC	::::::.::::::.:::: :	UAGUUCCUCCAAACGCUUCUA	Cleavage	179-199
mtr-miR395h	MtMCU5	3.5	AUGAAGUGUUUGGGGGAACUU	::::::.::::::.::::	UAGUUCCUCCAAACGCUUCUA	Cleavage	179-199
mtr-miR395i	MtMCU5	3.5	AUGAAGUGUUUGGGGGAACUC	::::::.::::::.::::	UAGUUCCUCCAAACGCUUCUA	Cleavage	179-199
mtr-miR395j	MtMCU5	3.5	AUGAAGUGUUUGGGGGAACUC	::::::.::::::.::::	UAGUUCCUCCAAACGCUUCUA	Cleavage	179-199
mtr-miR395k	MtMCU5	2	UUGAAGCGUUUGGGGGAACUC	::::::.::::::::::: :	UAGUUCCUCCAAACGCUUCUA	Cleavage	179-199
mtr-miR395l	MtMCU5	3.5	AUGAAGUGUUUGGGGGAACUC	::::::.::::::.::::	UAGUUCCUCCAAACGCUUCUA	Cleavage	179-199
mtr-miR395m	MtMCU5	3.5	AUGAAGUGUUUGGGGGAACUC	::::::.::::::.::::	UAGUUCCUCCAAACGCUUCUA	Cleavage	179-199
mtr-miR395n	MtMCU5	3.5	AUGAAGUGUUUGGGGGAACUC	::::::.::::::.::::	UAGUUCCUCCAAACGCUUCUA	Cleavage	179-199
mtr-miR395o	MtMCU5	3.5	AUGAAGUGUUUGGGGGAACUC	::::::.::::::.::::	UAGUUCCUCCAAACGCUUCUA	Cleavage	179-199
mtr-miR530	MtMCU5	3.5	UGCAUUUGCACCUGCACUUUC	.:::. ::::::::::::	GUUGGUGUUGGUGCAAAUGCA	Cleavage	1078-1098
mtr-miR172d-5p	MtMCU1.1	3	AGUGGAGCAUCAUCAAGAUUCACA	.:::: :::::::::..::	UUGCGAUCUCGAUGAUGCUUUACA	Cleavage	1311-1334
mtr-miR172d-5p	MtMCU1.2	3.5	AGUGGAGCAUCAUCAAGAUUCACA	: : .::::::: ::::::..::	UCUUGAUCUUGAAGAUGCUUUACA	Cleavage	1174-1197

Table S5 | The primers used in this study

Gene name	Gene ID	Forward primer (5'-3')	Reverse primer (5'-3')
MsMCU1.1	*MsG0580028752.01*	TTTCACAACAGCAACTGGCTT	TGAAGCTCCTTGCATCTCACA
MsMCU1.2	*MsG0380015087.01*	GCACATAAACAGGTGCGACG	GCGTAGCCTGTAACTAACCCG
MsMCU5	*MsG0180000180.01*	CGAAGTTCACGGTGGAGGAT	TCTCACACGATTCAACGCAGA
MsMCU6.1	*MsG0580024923.01*	AGGAGGATGGTGTTAGCGTC	GCCGCAGAATCATCAAGCAT
MsMCU6.2	*MsG0880045757.01*	GCCGTTTCAGCACTAAGCAG	TTCAACTCGGAAGAGGGTGC
MsActin	*MsG0380016789.01*	CCCACTGGATGTCTGTAGGTT	AGAATTAAGTAGCAGCGCAAA

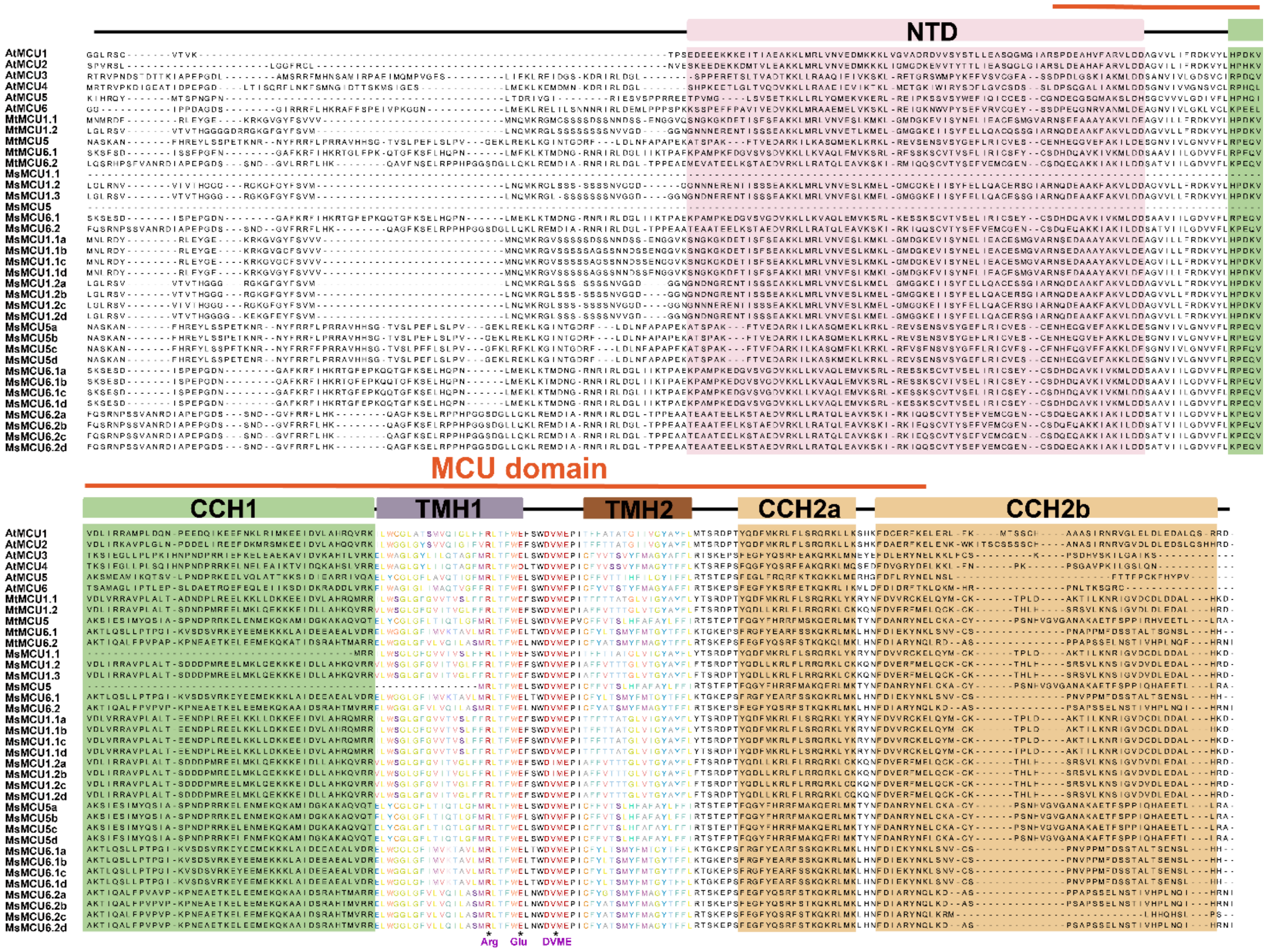

Supplemental Figure S1: Sequence alignment showing the conserved structure of MCU proteins.

NTD, N-terminal domain, CCH, coil-coil helix, TMH, transmembrane helix.

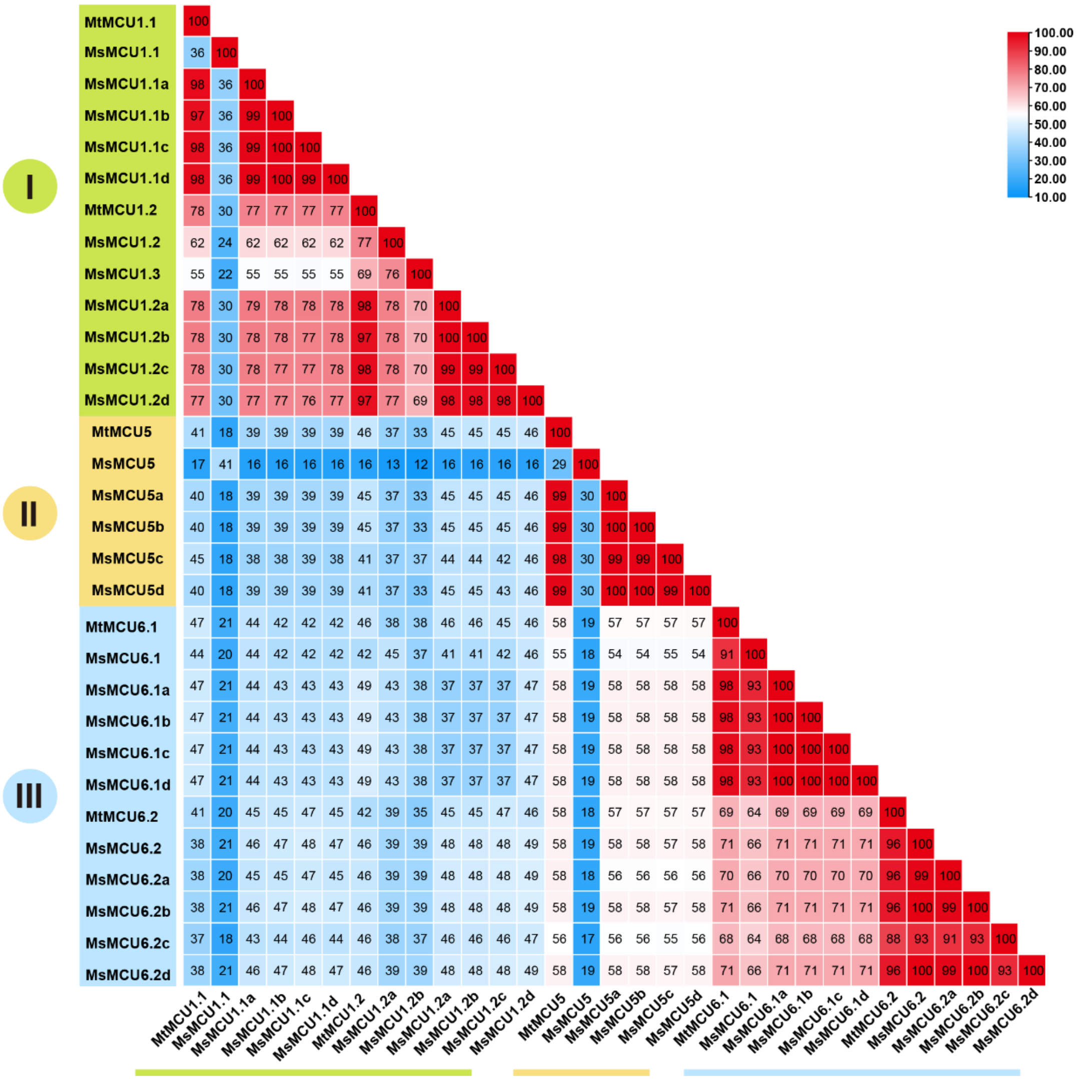

Supplemental Figure S2: Heatmap showing the similarity of MCUs proteins. The ruler is displayed in the right.

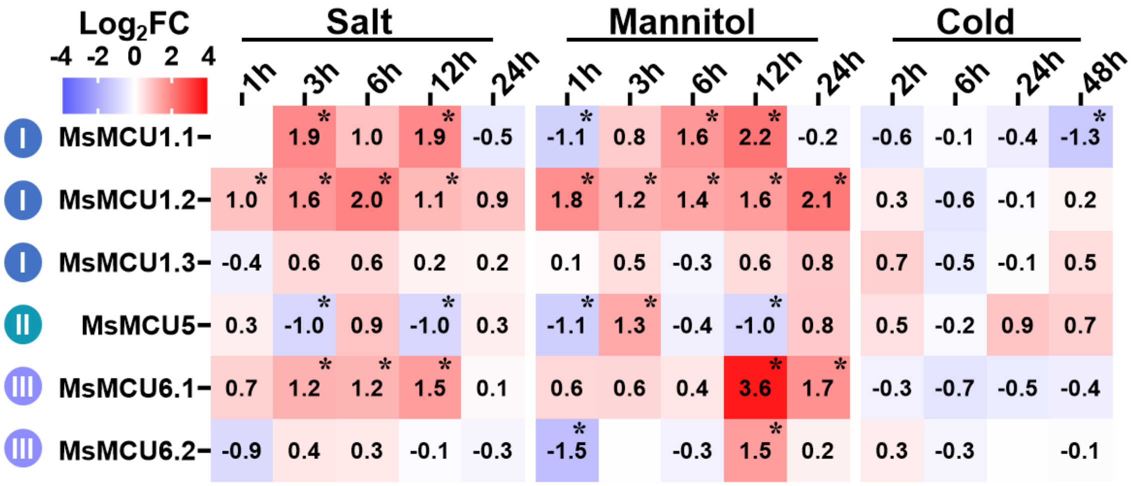

Supplemental Figure S3: Expression patterns of *MsMCUs* in response to salt, mannitol, and cold stresses. Numbers on the heatmap mean the FPKM values of each gene based on the transcriptome

data. * on the heatmap means differential expression by using the threshold $|\log_2 FC| \geq 1$. Roman numerals in colored circle dots represent different subfamilies of the MCU family.

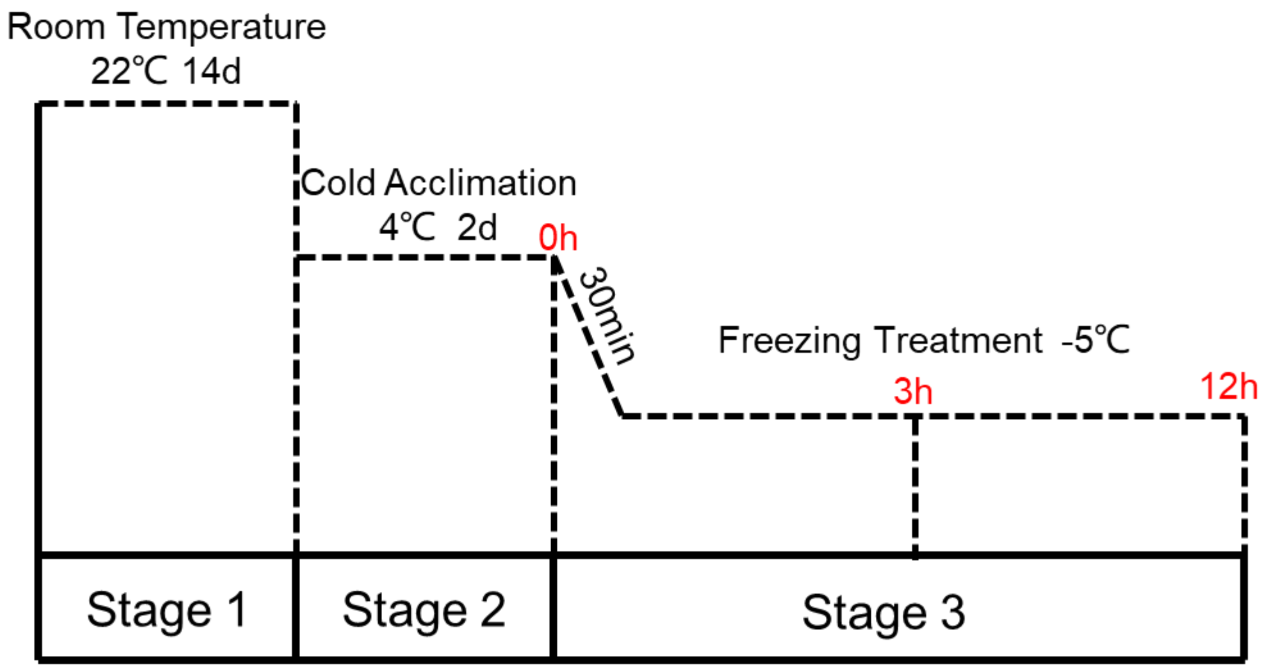

Supplemental Figure S4: Treatment methods for alfalfa seedlings under freezing stress. Seedlings grown under 22℃ for 14 days underwent cold acclimation at 4℃ for 2 days and then were subjected to freezing treatment at -5℃, and the samples were taken at 0h, 3h and 12h for gene expression level detection, respectively.
